# Transient expression of antinuclear RNP-A antibodies in patients with acute COVID-19 infection

**DOI:** 10.1016/j.jtauto.2022.100175

**Published:** 2022-11-24

**Authors:** Shuxia Zhou, Ravi Kaul, Kara L. Lynch, Alan H.B. Wu, Roger P. Walker

**Affiliations:** a Bio-Rad Laboratories, Hercules, CA, USA; b Department of Laboratory Medicine, University of California, San Francisco, USA

**Keywords:** SARS-CoV-2, COVID-19, Antinuclear antibody, RNP-A, Neutralizing antibody, Nucleocapsid protein, SARS-CoV-2, severe acute respiratory syndrome coronavirus 2, COVID-19, coronavirus disease 2019, ACE2, angiotensin-converting enzyme 2, ANA, antinuclear antibody, sVNT, surrogate viral neutralization test, Nab, neutralizing antibody, RNP-A, ribonucleoprotein A, MCTD, mixed connective tissue disease, ICU, intensive care unit, PSO, post symptom onset, N, SARS-CoV-2 nucleocapsid protein, IFA, Immunofluorescence assay

## Abstract

**Introduction:**

Viral infections have been implicated in the initiation of the autoimmune diseases. Recent reports suggest that a proportion of patients with COVID-19 develop severe disease with multiple organ injuries. We evaluated the relationship between COVID-19 severity, prevalence and persistence of antinuclear and other systemic and organ specific autoantibodies as well as SARS-CoV-2 infection specific anti-nucleocapsid (N) IgG antibodies and protective neutralizing antibody (Nab) levels.

**Methods:**

Samples from 119 COVID-19 patients categorized based on their level of care and 284 healthy subjects were tested for the presence and persistence of antinuclear and other systemic and organ specific autoantibodies as well as SARS-CoV-2 and neutralizing antibody levels.

**Results:**

The data shows significantly increased levels of anti RNP-A, anti-nucleocapsid and neutralizing antibody among patients receiving ICU care compared to non-ICU care. Furthermore, subjects receiving ICU care demonstrated significantly higher nucleocapsid IgG levels among the RNP-A positive cohort compared to RNP-A negative cohort. Notably, the expression of anti RNP-A antibodies is transient that reverts to non-reactive status between 20 and 60 days post symptom onset.

**Conclusions:**

COVID-19 patients in ICU care exhibit significantly higher levels of transient RNP-A autoantibodies, anti-nucleocapsid, and SARS-CoV-2 neutralizing antibodies compared to patients in non-ICU care.

## 1 Introduction

There is a strong association between viral infections and autoimmune diseases although the underlying etiology is not fully understood. Shoenfeld et al. have elegantly demonstrated that pathogenic viruses can trigger and initiate a host of autoimmune diseases [1,2]. Autoimmunity may manifest itself through molecular mimicry, bystander activation or epitope spreading [[3], [4], [5]]. The emergence of novel severe acute respiratory syndrome coronavirus 2 (SARS-CoV-2) poses a serious global public health threat that has infected over 590 million people globally with over 6.4 million deaths. Coronavirus disease 2019 (COVID-19) exhibits similarities to systemic autoimmune conditions including an association with increased incidence of autoantibodies [[6], [7], [8]]. It has been suggested that SARS-CoV-2 infection triggers a form of organ specific autoimmunity in predisposed patients [9]. Recently, Lerma et al. reported autoantibodies to nuclear antigens in 30% of SARS-CoV-2 patients, however, strong reactive autoantibodies were only detected in patients with prior history of autoimmune disease [8]. It is not clear from these studies whether any relationship exists between COVID-19 severity and the prevalence and persistence of autoantibodies. We describe the prevalence and transient expression of antinuclear antibodies, particularly anti-RNP-A autoantibodies, along with other systemic and organ specific autoantibodies in patients with mild to severe COVID-19 based on their level of care.

## 2 Materials and methods

### 2.1 Serum specimens

Remnant serum samples from 119 COVID-19 patients with positive RT-PCR results were collected from a clinical hospital laboratory between March 2020 and September 2021. All samples were de-identified to ensure patient confidentiality. Use of remnant samples from COVID-19 infected patients was approved by the University of California, San Francisco Institutional Review board (IRB protocol number 20–30387). Patient-reported symptom onset date and indicators of disease severity were extracted from electronic health records. Patients were categorized based on their level of care; patients admitted to an intensive care unit at any time during the disease course were classified as ICU patients, whereas those admitted to a hospital or managed as outpatients were considered non-ICU patients. Sera from 284 apparently healthy subjects were procured from commercial vendors. All samples were maintained at −20^0^C for the duration of the study. After thawing at room temperature, samples were briefly vortexed before testing in singlicate.

### 2.2 Autoantibody detection

All samples were tested by the BioPlex 2200 ANA screen assay that detects 13 IgG autoantibodies simultaneously against dsDNA, chromatin, ribosomal P, SSA-52, SSA-60, SSB, Sm, the Sm/RNP complex, RNP-A, RNP-68, Scl-70, centromere B, and Jo-1 within a single serum sample [10]. Serum samples of COVID-19 patients were tested for the presence of anti-cardiolipin, anti-β2GPI IgG, IgM and IgA isotype antibodies, anti-MPO, anti-PR3 and anti-GBM-IgG antibodies, anti-tTG and anti-Gliadin IgA and IgG antibodies, as well as anti-CCP IgG antibodies using the BioPlex 2200 anti-phospholipid syndrome (APLS) IgG, IgM and IgA, vasculitis panel IgG, gastrointestinal IgG and IgA as well as the BioPlex 2200 anti-CCP IgG kits. The BioPlex 2200 ANA reports an antibody index (AI) value in the range of 0.2–8.0 AI for all antibodies except anti-dsDNA for which IU/mL is used. The cutoff for the anti-dsDNA antibody is 10 IU/mL and for all other autoantibodies is 1.0 AI. Results are considered positive when there is at least one positive result for the antibodies detected by this panel. Patient samples with RNP-A positive results were confirmed by Kallestad Hep-2 ANA IFA kit at 1:40 and 1:80 titers.

### 2.3 Multiplex SARS-CoV-2 surrogate virus neutralization test (plex-sVNT)

The BioPlex 2200 sVNT assay is a bead-based multiplex assay that detects the presence of SARS-CoV-2 neutralizing antibodies in serum and/or plasma [11]. Essentially, neutralizing antibodies compete with biotinylated-human ACE2-Fc protein for binding to trimeric spike proteins that are coupled to beads. An assay cutoff of 25% inhibition for ACE2-trimeric spike protein binding was established based on 99th percentile cutoff using commercially available healthy normal, pregnancy and potential cross reactant samples.

### 2.4 BioPlex 2200 SARS-CoV-2 IgG assay panel

The BioPlex 2200 SARS-CoV-2 IgG assay is a multiplex assay that detects IgG antibodies against the receptor-binding domain (RBD), Spike 1 (S1), Spike 2 (S2), and nucleocapsid protein (N) of the SARS-CoV-2 virus. The assay is commercially available outside of the United States (OUS). Essentially, uniquely classified beads are coated with one of the four antigens independently and the amount of antibody captured by each antigen is determined by the fluorescence of the attached PE. Raw data was calculated in relative fluorescent intensity (RFI). The assay is calibrated using six distinct calibrator levels for each marker and semi-quantitative results expressed in U/mL using 4-PL curve fit. The presence of RBD, S1 and S2 IgG antibodies appear in infected as well as vaccinated uninfected subjects as opposed to the nucleocapsid antibodies that are predominantly present in infected subjects only.

### 2.5 Statistical analysis

Difference in antinuclear and other systemic and organ specific autoantibody prevalence levels were evaluated using Fisher's exact test where statistical significance was defined as p < 0.05. The differences in neutralizing SARS-CoV-2 antibodies and anti-N antibodies for patients with positive and negative RNP-A levels were assessed by two-tailed *t*-test, where statistical significance is defined as p < 0.05. Statistical analysis was performed using *GraphPad Prism* 9.0 (version 9.4.0).

## 3 Results

403 samples obtained from 284 apparently healthy subjects and 119 SARS-CoV-2 RT-PCR positive patients were included in this study. Of the 119 SARS-CoV-2 RT-PCR confirmed patients, 41 (34.5%) were admitted to the ICU while 78 (65.5%) patients were classified as non-ICU because they were either admitted to the hospital or managed as outpatients. Samples from patients receiving ICU care were collected an average of 23.3 days (7–88 days) post symptom onset while non-ICU samples were obtained 44.2 days (range 5–88 days) post symptom onset. Matched analysis between the two patient cohorts was restricted to samples collected up to 90 days post symptom onset to reduce the impact of confounding variables. While 10.2% (29/284) of the healthy population demonstrated antinuclear autoantibodies (ANA), the non-ICU patient cohort displayed a prevalence of 17.9% (14/78) compared to an exceptionally high prevalence of 43.9% (18/41) in the ICU cohort (Table 1). The majority of patients displayed reactivity to one target autoantigen: RNP-A. Only 3 of 78 non-ICU and 2 of 41 ICU samples demonstrated reactivity to more than one target antinuclear autoantibodies (data not shown). Compared to the healthy group with RNP-A prevalence of 3.9%, the non-ICU and ICU sample cohorts displayed significantly higher prevalence of 10.3% and 31.7% (p value 0.0401 and < 0.0001) respectively (Table 1). An overall increase in RNP-A autoantibodies among ICU patients compared to the non-ICU cohort suggests a progressive increase in antibody levels as a function of disease severity (Table 2). We also sought to determine whether ICU and non-ICU cohorts correlate with SARS-CoV-2 anti-N IgG antibody levels, a disease specific marker, as well as neutralizing antibody levels that prevent/protective against the disease. Our data shows that the means of both anti-N IgG and Nab levels are significantly different between the ICU and non-ICU patient cohorts (Table 2).

**Table 1:** Prevalence of antinuclear antibodies in COVID-19 patients and apparently healthy subjects.

Antinuclear	COVID-19 Patients			Apparently	p (healthy vs)	
antibody	non-ICU (N = 78)	ICU (N = 41)	p	Healthy (N = 284)	non-ICU	ICU
ANA	17.9% (14/78)	43.9% (18/41)	0.0042	10.2% (29/284)	0.0748	<0.0001
dsDNA	0.0% (0/78)	0.0% (0/41)	>0.9999	1.4% (4/284)	0.5813	>0.9999
Chromatin	2.6% (2/78)	2.4% (1/41)	>0.9999	0.7% (2/284)	0.2042	0.3336
RNP-A	10.3% (8/78)	31.7% (13/41)	0.0053	3.9% (11/284)	0.0401	<0.0001
SS-B	1.3% (1/78)	2.4% (1/41)	>0.9999	1.1% (3/284)	>0.9999	0.4185
SS-A52	1.3% (1/78)	4.9% (2/41)	0.2725	1.1% (3/284)	>0.9999	0.1214
Scl-70	1.3% (1/78)	0.0% (0/41)	>0.9999	1.4% (4/284)	>0.9999	>0.9999
Sm	0.0% (0/78)	2.4% (1/41)	0.3445	0.0% (0/284)	>0.9999	>0.9999
Cent B	1.3% (1/78)	2.4% (1/41)	>0.9999	0.0% (0/284)	>0.9999	>0.9999
SmRNP	0.0% (0/78)	4.9% (2/41)	0.1168	0.0% (0/284)	>0.9999	0.0156
Ribo P	0.0% (0/78)	0.0% (0/41)	>0.9999	0.4% (1/284)	>0.9999	>0.9999
RNP 68	1.3% (1/78)	2.4% (1/41)	>0.9999	0.0% (0/284)	>0.9999	>0.9999
SS-A60	1.3% (1/78)	0.0% (0/41)	>0.9999	1.4% (4/284)	>0.9999	>0.9999
Jo-1	0.0% (0/78)	0.0% (0/41)	>0.9999	0.0% (0/284)	>0.9999	>0.9999

Fisher's exact test, P < 0.05, significant difference.

**Table 2:** Comparison of anti-RNP-A, anti-N IgG and neutralizing antibody levels among COVID-19 patients requiring ICU and non-ICU care and RNP-A positive and negative patient cohorts.

Cohort	N	RNP-A (AI)	anti-N IgG (U/mL)	Nab inhibition (%)
ICU	41	1.2	1044.0	87.1%
non-ICU	78	0.4	192.8	58.6%
p value		0.0006	0.0002	<0.0001
RNP-A +	21	NA	1609.9	83.2%
RNP-A -	98	NA	234.0	63.2%
p value		NA	<0.0001	0.0031

*t*-test, p value < 0.05, significant difference.

The observation that both anti-N IgG and Nab levels reach statistical significance among the combined disease group (ICU and non-ICU) between RNP-A positive and RNP-A negative cohorts (Table 2) is suggestive of a relationship between disease severity and expression of RNP-A autoantibodies. At the same time, all other systemic and organ specific autoantibodies failed to exhibit any appreciable difference between the two diseased cohorts (Table 3). It is important to note that 3/41 PCR positive ICU patients and 5/78 non-ICU patients tested negative by the anti-N IgG assay. Lack of anti-N IgG antibodies in approximately 7% of PCR positive patients is probably due to late sero-conversion and/or higher sensitivity of RT-PCR assay.

**Table 3:** Prevalence of systemic and organ specific autoantibodies in COVID-19 patients receiving ICU and non-ICU care.

Autoantibodies	non-ICU	ICU	p*
ANA	17.9% (14/78)	43.9% (18/41)	0.0042
CCP	1.3% (1/78)	4.9% (2/41)	0.2725
β2-GP IgM	3.8% (3/78)	2.4% (1/41)	>0.9999
Cardiolipin IgM	3.8% (3/78)	2.4% (1/41)	>0.9999
β2-GP IgG	2.6% (2/78)	0.0% (0/41)	0.5445
Cardiolipin IgG	0.0% (0/78)	0.0% (0/41)	>0.9999
β2-GP IgA	3.8% (3/78)	0.0% (0/41)	0.5503
Cardiolipin IgA	5.2% (4/78)	2.4% (1/41)	0.6584
GBM	0.0% (0/78)	0.0% (0/41)	>0.9999
MPO	1.3% (1/78)	2.4% (1/41)	>0.9999
PR3	0.0% (0/78)	0.0% (0/41)	>0.9999
DGP IgA	3.8% (3/78)	4.9% (2/41)	>0.9999
tTG IgA	0.0% (0/78)	0.0% (0/41)	>0.9999
DGP IgG	2.6% (2/78)	0.0% (0/41)	0.5445
tTG IgG	0.0% (0/78)	7.3% (3/41)	0.0389
Multiple autoantibodies	10.3% (8/78)	24.4% (10/41)	0.0584
Overall autoantibodies	26.9% (21/78)	48.8% (20/41)	0.0251

Fisher's exact test, p < 0.05, significant difference.

**Fig. 1:**
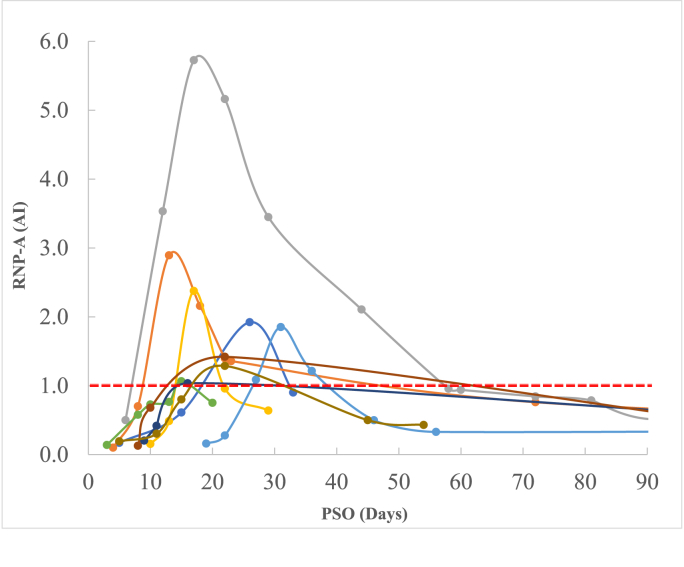
RNP-A seroconversion in ICU COVID-19 patients. The appearance and persistence of autoantibodies was plotted vs. the days since symptom onset. Each donor is shown in a different color. Exceptionally high RNP-A antibodies (>8.0 AI) were observed for one sample for which no additional sample draws were available. One sample was excluded from this figure because all three blood draws exceeded the assay range. Follow up draws from this patient were not available. Dotted line represents assay cutoff level of 1.0 AI.

**Fig. 2:**
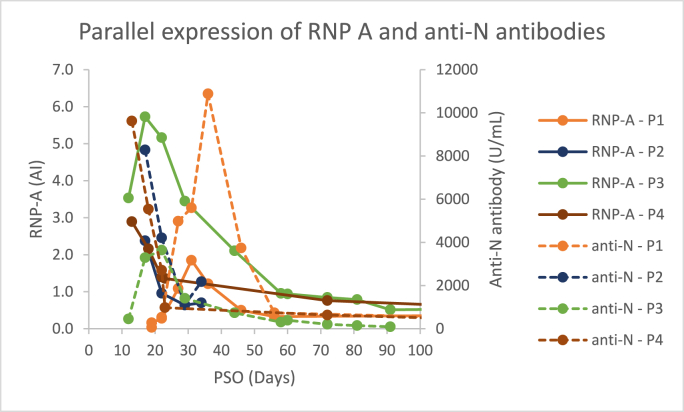
Temporal profiles of four representative anti RNP-A and anti-N antibodies among patient samples labeled P1 – P4 with serial draws.

**Fig. 3:**
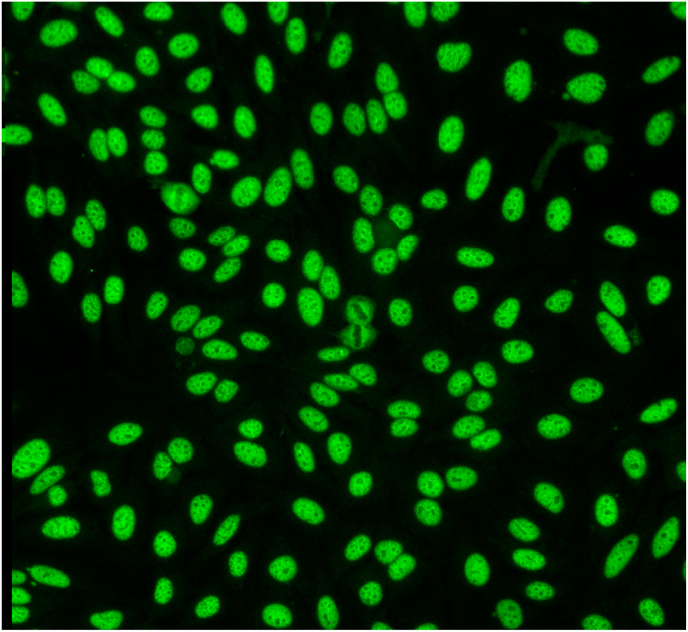
Representative IFA ANA patterns on HEp-2 cells. Anti-RNP-A antibody positive samples demonstrated a typical coarse speckled pattern at 1:80 titer.

Production and persistence of autoantibodies against RNP-A was examined by analyzing multiple blood draws from 10 ICU patients with positive reactivity. Nine out of ten patients sero-converted reaching peak levels between 13 and 31 days post-symptom onset. Of these nine sero-conversion samples, six displayed RNP-A peak levels between 2 and 6 times the assay cutoff levels. Nonetheless, sero-conversion proved transient because all nine samples reverted to non-reactive status between 20 and 60 days post-symptom onset (Fig. 1). Of the nine RNP-A positive samples, four patients also demonstrated transient expression of other autoantibodies including anti-MPO IgG, anti-tTG IgG and anti-CCP antibodies. Importantly, temporal profiles of RNP-A among serial draws parallel anti-N IgG antibody expression levels, although the levels of anti-N antibody never became negative (Fig. 2). In contrast, other systemic and organ specific autoantibodies failed to exhibit any appreciable change with disease progression. Next, we evaluated ANA in RNP-A positive samples using an IFA assay as a confirmatory test. All RNP-A positive samples were confirmed positive for ANA autoantibodies by IFA using Kallestad HEp-2 substrate at 1:40 and 1:80 titers. All samples demonstrated nuclear speckled pattern as shown in the slide image (Fig. 3). One patient with three blood draws taken between days 27–60 post symptom onset maintained off-scale levels (>8.0 AI) and positive IFA results. No additional blood draws were available for this patient.

## 4 Discussion

Recent work has demonstrated increased prevalence of anti-nuclear antibodies in acute COVID-19 patients, however, these studies failed to demonstrate an association between disease severity, autoantibody expression and long-term persistence (6–8). We compared the prevalence of anti-nuclear and other systemic and organ specific autoantibodies among acute (ICU care), mild (non-ICU care) COVID-19 patients, and apparently healthy populations. We report progressive increases in anti-nuclear and more specifically anti-RNP-A autoantibody, COVID-19 specific anti-N antibody and protective Nab levels among patients receiving ICU care vs. non-ICU care. These observations lend credence to the argument that disease severity plays a role in increased prevalence of anti-RNP-A antibodies; however, the underlying mechanism is far from clear. None of the patients in the ICU or non-ICU setting were previously diagnosed with autoimmune disorder. It has been reported that ANA positive patients had a poor prognosis compared to the negative patients with regards to COVID-19 disease [12,13]. We could not confirm these findings using hospital stay as a criterion. Indeed, the RNP-A autoantibody positive ICU patients displayed longer hospitalization times; but this parameter didn't reach statistical significance compared to the RNP-A negative ICU patients.

Garcia-Beltran [14] reported significantly diminished neutralizing potency in severely ill patients. We evaluated neutralizing antibody levels among ICU and non-ICU patients. Significantly higher levels of neutralizing and anti-N antibodies levels were observed in the ICU group. The prevalence of ANA and RNP-A antibodies in healthy populations and infectious disease patients has been reported extensively [[15], [16], [17], [18]]. RNP-A is one of the three RNP autoantigens (called A, C and 68 kD) located in the cell nucleus. High antibody titers to nuclear ribonuclear protein is suggestive of mixed connective tissue disease (MCTD), especially in the absence of other autoimmune antibodies such as anti-Smith, anti-SSA/ro and SSB/la antibodies [19]. According to the Alarcon-Segovia criteria, MCTD is diagnosed with an RNP antibody titer of >1:1600 and ≥ 3 clinical criteria, including synovitis, myositis, edema in hands, Raynaud phenomenon, and arcosclerosis [20]. Although the RNP titers of these patients met these criteria, unfortunately the clinical criteria could not be assessed. The pathophysiology mechanism for the release of RNP antibody during a SARS-CoV-2 infection is unknown. The fact that we observed a higher incidence of these antibodies for patients admitted to an ICU vs. hospital admission or outpatients suggest that these antibodies instead may play a harmful role during the infection. Excessive release of cytokines and various autoimmune antibodies have been well described in patients with serious COVID-19 infections [21]. Ahmed et al. [22] have suggested that patients with pre-existing rheumatic diseases may flare during the SARS-CoV-2 infection. However, none of the RNP-A positive COVID-19 patients in this study revealed MCTD or other systemic autoimmune diseases. Coupled with these observations is the fact that differences in other systemic and organ specific autoantibody levels never reached statistical significance between ICU and non-ICU patient cohorts. Whether this is due to the small cohort size remains to be explored.

RNP-A seroconversion panels serve as a valuable tool for investigating immune responses. It has been argued that autoimmune responses may develop through virus induced hyper-stimulation of the immune system or alternatively through molecular mimicry due to resemblance between the virus and the host [23]. It is also possible that amino acid sequences contained within SARS-CoV-2 resemble other sequences present within human proteins to illicit a mimicry immune response. As viral proteins are cleared from the circulation from a recovering infection patient, so too could the stimulus for autoantibody production. Neutralization of HIV type I infectivity by serum antibodies from a subset of autoimmune patients with mixed connective tissue disease was demonstrated in earlier studies [24]. Whether a similar mechanism prevails among COVID-19 patients due to retroviral nature of the virus, was outside the scope of this study, though early recovery using hospital stay as a criterion would not support this conclusion. The fact that nine out of ten ICU patients demonstrated transient RNP-A seroconversion on consecutive sample draws suggests direct sequalae of COVID-19, however, long-term consequences of SARS-CoV-2 infection in recovered patients need to be determined. Transient expression of antinuclear antibodies has been reported for other medical conditions as well [25]. It has been suggested that the risk of developing or increasing the autoimmune response may enhance and adversely impact the outcome of COVID-19 patients [26]. Whether patients with transient RNP-A develop long COVID or new autoimmune manifestations is unknown at this time.

## Author contributions

SZ contributed to conducting the study, analyzing data, and writing the manuscript. RK contributed to study design and writing the manuscript. KLL and AHBW contributed to the patient sample and clinical information acquisition. RPW and AHBW reviewed and edited the manuscript. All authors have approved the manuscript.

## Declaration of competing interest

The authors declare that they have no known competing financial interests or personal relationships that could have appeared to influence the work reported in this paper.

## Data Availability

Data will be made available on request.

## References

[1] Shoenfeld Y., Isenberg D.A. (1989). The mosaic of autoimmunity. Immunol. Today.

[2] Amital H., Gershwin E.M., Shoenfeld Y. (2006). Reshaping the mosaic of autoimmunity. Semin. Arthritis Rheum..

[3] Fujinami R., von Herrath M.G., Christen U. (2006). Molecular mimicry, bystander activation or viral persistence: infections and autoimmune disease. Clin. Microbiol. Rev..

[4] Ercolini A.M., Miller S.D. (2009). The role of infections in autoimmune disease. Clin. Exp. Immunol..

[5] Hussein H.M., Rahal E.A. (2019). The role of viral infections in the development of autoimmune diseases. Crit Rev Microbiol 45.

[6] Chang S.E., Minn D., Kim Y.K. (2021). Autoantibodies in moderate to critical cases of COVID 19. Clin Transl Sci.

[7] Muratori P., Lenzi M., Muratori L., Granito A. (2021). Antinuclear antibodies in COVID19. Clin Transl Sci.

[8] Lerma L.A., Chaudhary A., Bryan A., Morishima C., Wener M.H., Fink S.L. (2020). Prevalence of autoantibody response in acute coronavirus disease 2019 (COVID 19). J Transl Autoimmun.

[9] Gagiannis D., Steinestel J., Hackenbroch C. (2020).

[10] Shovman O., Gilburd B., Barzilai O., Shinar E., Larida B., Zandman-Goddard G., Binder S.R., Shoenfeld Y. (2005). Ann N Y Acad Sci. 6.

[11] Lynch K.L., Zhou S., Kaul R., Walker R., Wu A.H.B. (2022). Evaluation of neutralizing antibodies against SARS-CoV-2 variants after infection and vaccination using a multiplexed surrogate virus neutralization test. Clin. Chem..

[12] Pascolini S., Vannini A., Deleonardi G. (2021). COVID-19 and immunological dysfuncion: can autoantibodies be useful?. Clin Trans Sci.

[13] Liu Y., Sawalha A.H., Lu Q. (2020). COVID-19 and autoimmune disease. Curr. Opin. Rheumatol..

[14] Garcia-Beltran W.F., Lam E.C., Astudillo M.G., Yang D., Miller T.E., Feldman J., Hauser B.M., Caradonna T.M., Clayton K.L., Nitido A.D., Murali M.R., Alter G., Charles R.C., Dighe A., Branda J.A., Lennerz J.K., Lingwood D., Schmidt A.G., Iafrate A.J., Balazs A.B. (2021). COVID-19-neutralizing antibodies predict disease severity and survival. Cell.

[15] Shovman O., Gilburd B., Barzilai O., Shinar E., Larida B., Zandman-Goddard G. (2005). Evaluation of the BioPlex™ 2200 ANA screen: analysis of 510 healthy subjects: incidence of natural/predictive autoantibodies. Ann. N. Y. Acad. Sci..

[16] Katz I., De Luca F., Dzudor B., Sarpong B.K., Osei-Appiah B., Azoulay D. (2020). Seroprevalences of autoantibodies and anti-infectious antibodies among Ghana's healthy population. Sci. Rep..

[17] Litwin C.M., Rourk A.R. (2018). Anti-ENA antibody profiles in patients with hepatitis C virus infection. J. Clin. Lab. Anal..

[18] Yuan W., Cao H., Li W., Wu X., Zheng J. (2022). Comparison study of bead-based and line-blot multiplex ANA immunoassays in the diagnosis of systemic autoimmune rheumatic diseases. Clin. Rheumatol..

[19] Combe B., Rucheton M., Graafland M., Lussiez V., Brunel C., Sany J. (1989). Clinical significance of anti-RNP and anti-Sm autoantibodies as determined by immunoblotting and immunoprecipitation in sera from patients with connective tissue disease. Clin. Exp. Immunol..

[20] Alacon-Segovia D., Cardiel M.H. (1989). Comparison between three diagnostic criteria for mixed connective tissue disease. Study of 593 patients. J. Rheumatol..

[21] Zuo Y., Estes S.K., Ali R.A., Gandhi A.A., Yalavarthi S., Shi H. (2020). Prothrombotic autoantibodies in serum from patients hospitalized with COVID-19. Sci. Transl. Med..

[22] Ahmed S., Zimba O., Gasparyan A.Y. (2021). COVID-19 and the clinical course of rheumatic manifestation. Clin. Rheumatol..

[23] Dotan A., Muller S., Kanduc D., David P., Halpert G., Shoenfeld Y. (2021). The SARS-CoV2 as an instrumental trigger of autoimmunity. Autoimmun. Rev..

[24] Douvas A., Takehana Y., Ehresmann G., Chernyovskiy T., Daar E.S. (1996). Neutralization of HIV Type I infectivity by serum antibodies from a subset of autoimmune patients with mixed connective tissue disease. AIDS Res. Hum. Retrovir..

[25] Koshy M., Mathew J., Alex R., Jude J.A., Ralph R., Sudarsanam T.D., Sathyendra S., Visalakshi J., Peter J.V. (2018). Antinuclear antibodies in scrub typhus: transient occurrence during acute illness. J. Vector Borne Dis..

[26] Sacchi M.C., Tamiazzo S., Stobbione P., Agatea L., Gaspari P.D., Stecca A., Lauritano E.C., Roveta A., Tozzoli R., Guaschino R., Bonometti R. (2021). SARS-CoV-2 infection as a trigger of autoimmune response. Clin Transl Sci.

